# Birth weight concerning obesity and diabetes gene expression in healthy infants; a case-control study

**DOI:** 10.1186/s12884-023-05538-0

**Published:** 2023-03-30

**Authors:** Sahar Cheshmeh, Shima Moradi, Seyyed Mostafa Nachvak, Arman Mohammadi, Nastaran Najafi, Azadeh Erfanifar, Arezoo Bajelani

**Affiliations:** 1 Molecular and Experimental Nutritional Medicine Department, University of Potsdam, Nuthetal, Germany; 2 Student Research Committee, Kermanshah University of Medical Sciences, Kermanshah, Iran; 3 Department of Nutritional Sciences, School of Nutritional Sciences and Food Technology, Kermanshah University of Medical Sciences, Kermanshah, Iran; 4 Department of Nutritional Sciences, Research Center for Environmental Determinants of Health (RCEDH), Health Institute, Kermanshah University of Medical Sciences, Kermanshah, Iran; 5 PhD Student, Student Research Committee, Iran University of Medical Sciences, Tehran, Iran; 6 PhD student, Nursing and Midwifery Care Research Center, Iran University of Medical Science, Tehran, Iran; 7 Cellular and Molecular Research Center, Cellular and Molecular Medicine Research Institute, Urmia University of Medical Sciences, Urmia, Najafi Iran; 8 Department of Nutrition, School of Paramedicine, Jundishapur University of Medical Science, Ahvaz, Iran; 9 Exercise Physiology Faculty of Human Sciences and Research Campus, Azad Islamic University, Tehran, Iran

**Keywords:** Low birth weight, Obesity, Diabetes, Gene expression

## Abstract

**Background:**

Since obesity and diabetes are prevalent worldwide, identifying the factors affecting these two conditions can effectively alter them. We decided to investigate the expression of obesity and diabetes genes in infants with birth weights lower than 2500 g in comparison with infants with normal birth weights.

**Methods:**

215 healthy infants between the ages of 5–6 months were used in the current case-control research, which was conducted at health and treatment facilities in Kermanshah. Infants who were healthy were chosen for the research after their weight and height were measured and compared to the WHO diagram to ensure that they were well-grown and in good health. There were 137 infants in the control group and 78 infants in the case group. All newborns had 5 cc of blood drawn intravenously. To assess the expression of the genes *MC4R*, *MTNR1B*, *PTEN*, *ACACB, PPAR-γ, PPAR-α*, *NRXN3*, *NTRK2*, *PCSK1*, *A2BP1*, *TMEM18*, *LXR*, *BDNF*, *TCF7L2*, *FTO* and *CPT1A*, blood samples were gathered in EDTA-coated vials. Chi-square, Mann-Whitney U, and Spearman analyses were used to examine the data.

**Results:**

A significant inverse correlation between birth weight and obesity and diabetes genes, including *MTNR1B, NTRK2, PCSK1*, and *PTEN* genes (r= -0.221, -0.235, -0.246, and − 0.418, respectively). In addition, the LBW infant’s expression level was significantly up-regulated than the normal-weight infants (P = 0.001, 0.007, 0.001, and < 0.001, respectively). The expression level of the *PPAR-a* gene had a significantly positive correlation with birth weight (r = 0.19, P = 0.005). The expression level of the *PPAR-a* gene in the normal-weight infants was significantly up-regulated than the LBW infants (P = 0.049).

**Conclusion:**

The expression levels of *MTNR1B, NTRK2, PCSK1*, and *PTEN* genes were up-regulated in the LBW infants; however, the expression level of *PPAR-a* gene was significantly down-regulated in the LBW infants compared to the infants with normal birth weight.

## Introduction

According to the data published by the Centers for Disease Control and Prevention (CDC), the prevalence of obesity in the American population is estimated to be 40% in 2022 [1]. The increasing prevalence of obesity in recent years in the worldwide has turned it into a major challenge for health systems and especially in children [2]. Moreover, type 2 diabetes has been developing resulting in childhood obesity which may contribute to increasing in non-communicable diseases [3].

Low birth weight (LBW) is enhanced short-term and long-term consequences such as infant mortality, cognitive disorders, and growth failure, and is associated with obesity and diabetes in adulthood [4, 5]. According to the definition by World Health Organization, LBW infants refer to infants whose birth weight is less than 2500 g [6]. Based on the UNICEF-WHO reports, approximately 15–20% of births are related to LBW infants which annually account for about 20 million births in worldwide, and about half of them are in South Asia [7]. Some studies have found a U-shaped or J-shaped association between birth weight and obesity in adulthood, although these findings have been contradictory [8, 9]. On the other hand, it has been suggested that LBW can increase the risk of developing diabetes in adulthood [8]. Since obesity and diabetes are prevalent worldwide; identifying the factors affecting these two conditions can effectively modify them [10, 11].

Both environmental and genetic factors are involved in the pathogenesis of obesity and diabetes [11–13]. The relationship between obesity and the human genome map and biological pathways involved in pathogenesis is limited [12]. Studies on twins and families have reported the heritability of obesity to be between 40% and 70% [12, 14]. As a result, the knowledge of genetic approaches effective in obesity can be used to define the basic physiological and molecular mechanisms that control body weight [14]. Furthermore, genetic factors play an important role in determining a person’s tendency to gain weight [15]. Among the genes involved in the etiology of obesity and diabetes can be named Melanocortin 4 receptor (*MC4R*), Melatonin Receptor 1B (*MTNR1B*), Peroxisome proliferator-activated receptor gamma (*PPAR-γ*), Phosphatase and tensin homolog (*PTEN*), Acetyl-CoA Carboxylase Beta (*ACACB*), and peroxisome proliferator-activated receptor-α (*PPAR-α*), *MC4R* gene encodes melanocortin receptor, MC_4_ protein, a G-protein receptor that high it’s expression is related to body fat distribution and energy intake in children [16]. *MTNR1B* gene is located on chromosome 11q21 and synthesis melatonin receptor 2 in which this gene is related to all diabetes types including diabetes mellitus, type 1 diabetes, and gestational diabetes [17, 18]. In addition, down- regulated of *PTEN* and *ACACB* genes can decrease blood sugar and subsequently prevent diabetes and obesity *PTEN* is a phosphatase which plays role in signaling pathway and suppression of tumor [17, 19]. *ACACB* is a biotin dependent enzyme which catalyzes irreversible carboxylation of acetyl CoA to manolyl CoA and is effective in obesity and diabetes by reducing fatty acids oxidation and increasing of insulin resistance [20, 21]. Peroxisome proliferative activating receptors (*PPARs*) are part of the nuclear hormone receptors [22]. The up-regulated expression level of *PPAR-γ* regulates the secretion of adipose tissue hormones and reduces insulin resistance [23]. In addition, *PPAR-α* is expressed mostly in tissues with a high level of fatty acid catabolism. Thus, up-regulated *PPAR-α* reduces obesity and body fat [24]. According to the role of genetic factors on obesity and diabetes etiology, this study aimed to investigate the expression of obesity and diabetes genes in infants with birth weights lower than 2500 g in comparison with in infants with normal birth weight.

## Methods and materials

The current case-control study was performed on healthy infants aged 5–6 months referred to health and treatment centers in Kermanshah. The sample size was calculated based on the weighted mean of children in the study by Zarrati et al. [25] using the sample size formula for case studies with 90% power and 95% confidence in each group of 45 infants. Inclusion criteria were healthy infants, lack of metabolic diseases, with a weight range of 800–4000 g, not using medications last month, breastfeeding or using infant milk (formula), not initiating supplementary nutrition, healthy parent, and absence of metabolic illness of their parents (such as gestational diabetes), and did not have any drug addiction. Based on birth weight, we considered infants with a birth weight of less than 2500 g in the case group and weight between 2500 and 4000 g in the control group [6]. For more reassurance, we entered 80 infants in the case group and twice as many in the control group. Since some samples’ information was incomplete, therefore, 5 infants were excluded. Overall, 78 infants remained in the case group and 137 infants in the control group.

### Ethical consideration

Initially, the parents were given a thorough explanation of the study’s procedures and were asked for their written permission after being fully informed. This research from Kermanshah University of Medical Science was approved by the ethics committee (ethical number: IR.KUMS.RES.1397.081).

### Anthropometry

Firstly the health information of infant including term or preterm, birth height, weight, and head circumference, and the food kind was recorded in the questionnaire. We considered birth weight lower than 2500 g as LBW and birth weight between 2500 and 4000 g as normal weight. After that, the infants’ height was measured by tape and in supine position and the infants weight was measured with the least cloth and without diaper. After measuring weight and height of the infant, the values were compared with the diagram of WHO to assure being healthy and well-grown and infants who were healthy, were selected to the study. According to the WHO growth diagram, children whose height and weight growth percentiles were between 3 and 85 were selected as normal.

### Sampling and expression of obesity and diabetes genes

5 cc of intravenous blood was collected from all infants. The blood sample was then centrifuged at 500 rpm for 15 min. The serum isolated from the blood sample was frozen at minus − 80 °C. Blood samples were collected in Ethylenediaminetetraacetic acid (EDTA) coated vials to evaluate the expression of *MC4R*, *MTNR1B*, *PTEN*, *ACACB, PPAR-γ, PPAR-α*, Neurexin-3-alpha (*NRXN3*), Neurotrophic Receptor Tyrosine Kinase 2 (*NTRK2*), Proprotein convertase 1(*PCSK1*), Ataxin-2 binding protein 1 (*A2BP1*), Transmembrane 18 gene (*TMEM18*), Liver X receptor (*LXR*), Brain-derived neurotrophic factor (*BDNF*), transcription factor 7-like 2 (*TCF7L2*), fat mass and obesity-associated (*FTO*), and carnitine palmitoyltransferase IA (*CPT1A*) genes. The Ficoll-Histopaque solution gradient isolated peripheral blood mononuclear cells (PBMC) during density gradient centrifugation (Ficoll-paque, Miltenyi Biotec GmbH, and Germany). The Trisor Regaent kit extracted total RNA from PBMC cells (Iranian pure YTzol RNA). According to the manufacturer’s instructions, one microgram of extracted RNA was applied for complementary DNA synthesis (cDNA) by Prime Script-RT Reagent kits (Takara Bio Ink. Tokyo, Japan). Dedicated primers were purchased and designated from Metabion (Metabion, Germany) (Table 1). Data were normalized as housekeeping genes by 2-tCt expressing 18s-rRnan. All samples were done in three versions.

**Table 1:** Primers sequences for RT-PCR amplification

Gene name and symbol Sequence (5'→3')		Gene name and symbol Sequence (5'→3')	
*MC4R*	F: 5'-CTG ATG GAG GGT GCT ACG AG-3'	***BDNF***	F :5-GGCTTGACATCATTGGCTGAC-3'
	R :5'-TGG GTG AAT GCA GAT TCT TGT T-3'		R :5-TGTGCAGTGTGAGAAAGGCTT-3'
*MTNR1B*	F : 5'-GCA TGG CCT ACC ACC GAA TC-3'	***ACACB***	F : 5-CAAGCCGATCACCAAGAGTAAA-3'
	R : 5'-AAT AGA TGC GTG GGT CGT ACT-3'		R : 5-CCCTGAGTTATCAGAGGCTGG-3'
*TMEM18*	F : 5'-TGT TAA AGT CGA TGG TGT AGC TC-3'	***PTEN***	F : 5-CAAGATGATGTTTGAAACTATTCCAATG-3'
	R : 5'-GTC CTT GTC CGG TTG TGA ACT-3'		R : 5-CCTTTAGCTGGCAGACCACAA-3'
*TCF7L2*	F : 5'- CGGCGAGTCTATGCCACTAT-3'	***LXR-α***	F :5-CCTTCAGAACCCACAGAGATCC-3'
	R : 5'-ACACAGGGACCGAGTAATGC-3'		R :5-ACGCTGCATAGCTCGTTCC-3'
*NRXN3*	F : 5'-AGG GGA AAA TTG GAG TTG TCT TC-3'	***PPAR-γ***	F :5-GATGCCAGCGACTTTGACTC-3'
	R : 5'-CCG TCA TTT ACA GGG GTT CTC T-3'		R :5-ACCCACGTCATCTTCAGGGA-3'
*NTRK2*	F: 5'-ACC CGA AAC AAA CTG ACG AGT − 3'	***FTO***	F :5-ACTTGGCTCCCTTATCTGACC-3'
	R: 5'-AGC ATG TAA ATG GAT TGC CCA-3'		R :5-TGTGCAGTGTGAGAAAGGCTT-3'
*PCSK1*	F: 5'-ACC CGA AAC AAA CTG ACG AGT − 3'	***CPT1A***	F :5-TCCAGTTGGCTTATCGTGGTG-3'
	R: 5'-AGC ATG TAA ATG GAT TGC CCA-3'		R :5-TCCAGAGTCCGATTGATTTTTGC-3'
*A2BP1*	F: 5'-ATTCAAACTACTGCCACC-3'-3'	***PPAR-a***	F :5-ATGGTGGACACGGAAAGCC-3'
	R: 5'-TGTCTAACACCATCTGCTT-3'		R :5-CGATGGATTGCGAAATCTCTTGG-3'
*18s rRNA*	F :5'-ACCCGTTGAACCCCATTCGTG A-3'	F, forward	
	R :5'-GCCTCACTAAACCATCCAATCGG-3'	R, reverse	

### Statistical analysis

All variables in the current research were examined using SPSS (SPSS Inc. Chicago, IL, USA version 19) and STATA (Stata Corp, College Station, TX, version 14). The data normality was checked using Kolmogorov-Smirnov test. Basic characteristics of infants are described by mean, frequency, and per cent frequency. The Chi-square test was used to compare qualitative variables. Mann-Whitney U, Spearman correlation, and linear regression tests were used to evaluate the expression of obesity and diabetes genes in peripheral blood cells and their relationship with birth weight. A significance threshold of less than 0.05 was taken into consideration for all tests.

## Results

In this study, 137 infants and 78 infants were enrolled in the control and case study groups, respectively. In terms of the gender of the infants, the food kind of the infants, their mother’s age, their mother’s weight before pregnancy, and their mother and father’s BMI were no differences between the two studied groups. The mean weight in the control and case groups were 3.18 ± 0.27 and 1.79 ± 0.48 kg, respectively (P < 0.001). All characteristics of infants are presented in Table 2.

**Table 2:** Basic characteristics of infants based on the birth weight

Variables	Normal weight	Low birth weight	P1
	(n = 137)	(n = 78)	
Gender, boy %	54	51.3	0.403
Preterm, %	0	83.3	< 0.001
Food kind, %			
Breast feeding	34.3	30.8	0.09
Formula	35	24.4	
Mixed	30.7	44.9	
Birth weight, kg	3.18 ± 0.27*	1.79 ± 0.48	< 0.001
Birth height, cm	51.87 ± 3.71	42.92 ± 5.30	< 0.001
Head circumference, cm	33.69 ± 2.49	28.54 ± 2.85	< 0.001
Current weight, kg	7.87 ± 0.83	6.43 ± 1.4	< 0.001
Mother age, years	27.29 ± 5.86	28.41 ± 6.32	0.186
Mother weight before pregnancy, kg	69.7 ± 10.09	75.26 ± 13.13	0.359
Pregnancy weight gain, kg	14.18 ± 4.05	9.47 ± 8.63	< 0.001
Mother BMI, kg/m^2^	26.62 ± 4.09	25.74 ± 4.01	0.746
Father BMI, kg/m^2^	26.17 ± 3.14	26.12 ± 3.04	0.739
Mother smoking, %	2.2	0	0.257

*Mean ± SD

P1 was obtained Chi square and Mann-Whitney U Tests

The current study found a significant inverse correlation between birth weight and obesity and diabetes genes, including *MTNR1B, NTRK2, PCSK1*, and *PTEN* (r= -0.221, -0.235, -0.246, and − 0.418, respectively). In addition, the LBW infant’s expression level was significantly up-regulated than the normal weight infants (P = 0.001, 0.007, 0.001, and < 0.001, respectively) (Table 3).

**Table 3:** The expression level of obesity and diabetes genes in peripheral blood cells based on the birth weight

Obesity and diabetes genes	Spearman’s rho of birth weight		Normal weight (n = 137)	Low birth weight (n = 78)	P2	B (Unstandardized Coefficients)	Beta (Standardized Coefficients)	P3
	r	P1	Mean ± SD	Mean ± SD				
*MC4R*	0.079	0.288	13.27 ± 11.37*	16.54 ± 23.68	0.195	-0.001	-0.029	0.838
*MTNR1B*	-0.221	0.001	11.72 ± 11.90	22.83 ± 26.20	0.001	0.002	0.064	0.6
*NRXN3*	-0.011	0.873	6.93 ± 7.46	12.51 ± 18.71	0.4	-0.005	-0.092	0.534
*NTRK2*	-0.235	0.001	8.02 ± 10.52	15.48 ± 21.08	0.007	-0.006	-0.145	0.294
*PCSK1*	-0.246	0.001	6.23 ± 6.39	18.52 ± 24.03	0.001	0.004	0.077	0.578
*A2BP1*	-0.096	0.222	11.32 ± 8.34	20.58 ± 22.93	0.094	-0.004	-0.092	0.533
*TMEM18*	-0.027	0.717	10.19 ± 11.12	9.04 ± 9.22	0.76	0.022	0.278	0.033
*PPAR-γ*	-0.067	0.332	7.29 ± 10.79	11.90 ± 18.09	0.651	-0.005	-0.116	0.333
*LXR*	0.11	0.114	6.88 ± 8.92	4.19 ± 5.54	0.097	0.016	0.142	0.272
*PTEN*	-0.418	< 0.001	2.32 ± 2.82	49.16 ± 48.18	< 0.001	-0.009	-0.553	< 0.001
*ACACB*	-0.038	0.606	18.04 ± 20.08	22.37 ± 26.64	0.76	-0.013	-0.379	0.011
*BDNF*	-0.001	0.988	2.64 ± 2.92	2.56 ± 2.38	0.609	0.03	0.101	0.453
*TCFL2*	0.081	0.299	2.40 ± 2.74	2.42 ± 2.58	0.955	-0.049	-0.173	0.191
*FTO*	-0.06	0.396	12.81 ± 16.14	18.72 ± 23.11	0.142	-0.009	-0.177	0.223
*PPAR-a*	0.19	0.005	40.04 ± 17.62	21.51 ± 22.71	< 0.001	0	0.168	0.143
*CPT1A*	-0.104	0.142	22.19 ± 26.25	25.59 ± 25.45	0.161	0.011	0.347	0.017

P1 was obtained Spearman’s correlation

P 2 was obtained Mann-Whitney U

P3 was obtained Liner regression

Furthermore, in Table 3, we observed that the expression level of the *PPAR-a* gene had a significantly positive correlation with birth weight (r = 0.19, P = 0.005). The expression level of the *PPAR-a* gene in the normal weight infants was significantly up-regulated than the LBW infants (P = 0.049).

**Fig. 1:**
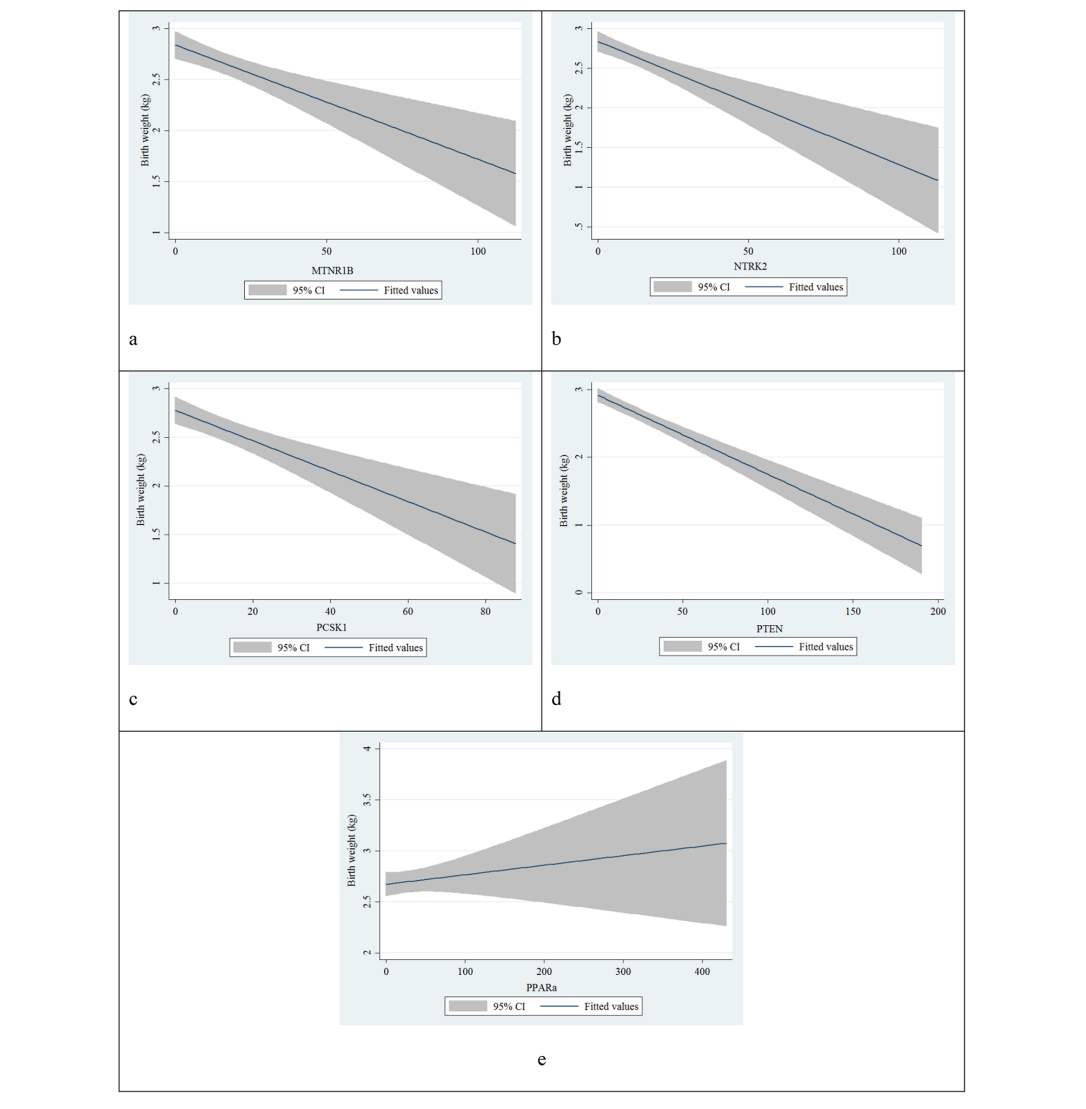
Linear regression between birth weight and obesity and diabetes genes including (**a**) *MTNR1B*, (**b**) *NTRK2*, (**c**) *PCSK1*, (**d**) *PTEN*, (**e**) *PPAR a*

Moreover, linear regression and graphs were applied to better showing these association the birth weight and these genes including *MTNR1B* (P = 0.6), *NTRK2* (P = 0.294), *PCSK1* (P = 0.578), *PTEN* (P < 0.001), and *PPAR-a* (P = 0.143) (Table 3; Fig. 1).

## Discussion

This present case-control study demonstrated overexpression of *MTNR1B, NTRK2, PCSK1*, and *PTEN* genes and down-regulation of *PPAR-a* gene in LBW. Birth weight is considered a suitable indicator for the quality of fetal growth and a predictor of health throughout life [26, 27]. Previous studies have indicated that LBW is associated with increased development of obesity, diabetes, neurodevelopment failure, cardiovascular disease, and other metabolic disorders [26]. Why does this happen? it is not clear. However, it seems that the decrease in birth weight is associated with a decrease in body hormone levels and changes in body composition, which is effective in the pathogenesis of obesity and diabetes in adulthood. Among these changes is the increase in leptin levels in the LBW infants, which seems to show a kind of resistance to leptin hormone in these infants [8, 28]. It has also been observed in animal studies that increased growth in these infants is mostly associated with fat cell hypertrophy and fat tissue dysfunction [29, 30]. Therefore, LBW increases fat absorption in childhood and subsequently adulthood [31]. Another possible effective mechanism was the decrease in the level of adiponectin hormone in some studies in the umbilical cord blood of LBW infants [32, 33]. However, since studies on the role of LBW on obesity and diabetes gene expression were limited, to the best of our knowledge, this current study evaluated the association between birth weight and obesity and diabetes gene expression in healthy infants.

Our findings indicated that birth weight was associated with the up-regulated expression level of *MTNR1B*. Holzapfel et al. [34] observed that *MTNR1B* was associated with diabetes in children and adolescents. Another study by Liang et al. [35] showed that maternal *MTNR1B* genotype is involved in the etiology of childhood obesity. Recently, *MTNR1B* has increased the risk of obesity and type 2 diabetes [34, 36]. It is also highly expressed in retinal cells, pancreas, and pancreatic islet cells. Melatonin is a neuro-hormone secreted by the pineal gland which can adjust the circadian rhythm and regulate the insulin level. However, melatonin secretion is impaired in diabetics [36].

We also indicated the up-regulated expression level of the *NTRK2* with increasing birth weight. In a study by Metrustry et al., variants in the *NTRK2* gene and birth weight were examined. This study showed this gene was highly expressed in LBW twins [37]. *NTRK2* is located on 9q21.33. *NTRK2* encodes a member of the neurotrophic tyrosine receptor kinase (NTRK) family, a membrane-bound receptor for *BDNF* and regulates energy balance downstream of *MC4R*. Also, it is involved (involves) in the MAPK pathway and cell differentiation [38]. Mutations of *NTRK2* have been associated with obesity and eating behavior [37, 39].

In the current study, the expression level of the *PCSK1* was up-regulated in LBW infants. Ruiz-Narváez et al. showed that LBW plays a role in the expression level of the *PCSK1* by disrupting central nervous system mechanisms and increasing obesity in adulthood [40]. *PCSK1*, located on 5q15 encodes a prohormone convertase 1/3 (PC 1/3) involved in pro-insulin processing under the influence of *TCF7L2* [41]. *PCSK1* is also engaged in processing pro-opiomelanocortin, proglucagon, proGnRH and proper. In addition, *PCSK1* variants are associated with extreme obesity, impaired glucose tolerance, and polycystic ovarian syndrome. Rare mutations in *PCSK1* cause childhood obesity, impaired pro-hormone processing and abnormal glucose homeostasis with increasing pro- insulin concentrations [40, 42].

In the present study, another up-regulated gene expression level was related to the *PTEN* gene. Li et al. showed that [43] the high expression level of gene *PTEN* was associated with increased insulin resistance. Although the gene was first identified as a tumor suppressor, it has recently been shown to be important with its antagonistic function in the insulin signaling cascade and is involved in glucose metabolism [44]. *PTEN* is a phosphatase that plays a role in the signaling pathway and tumor suppression in which it can suppress phosphatidylinositol 3-kinase (PI3K) signaling [17, 19, 45]. Since activation of PI3K is essential for insulin performance, *PTEN* is known to be effective in developing insulin resistance by reducing PI3K [46].

On the other hand, we observed that the expression level of the *PPAR-a* gene was significantly down-regulated in the LBW infants. Laleh et al. [47] demonstrated that the high expression level of *PPAR-a* suppressed appetite in obesity. Priego et al. [48] showed that the higher expression level of the *PPAR-α* gene in infants is associated with a lower risk of being overweight. *PPAR-a* is a group of legend activated nuclear receptors that are mainly expressed in tissues that are vital for fatty acid metabolisms, such as the liver, kidney, and heart, where they play an important role in regulating transcription of fatty acid metabolism, lipid homeostasis, and regulation of obesity [49]. During starvation and energy depletion, the *PPAR-α* increases fatty acid beta-oxidation. An animal study showed that *PPAR-a* deficiency was related to obesity and dyslipidemia [24].

### Limitations

This is the first case-control investigation into healthy infants’ birth weight, obesity, and diabetes gene expression. It was, however, constrained in some ways. The sample number was small because of financial limitations. The second, difference between the two groups was that many of the infants in the case group were preterm in terms of birth weight, pregnancy weight gain, and current weight infants, despite the fact that we controlled many infants and maternal variables between the two groups.

## Conclusions

In conclusion, this present study reflected that the expression level of *MTNR1B, NTRK2, PCSK1*, and *PTEN* genes were up-regulated in the LBW infants; however, the expression level of *PPAR-a* gene was significantly down-regulated in the LBW infants compared to the infants with normal birth weight.

## Data Availability

The datasets used and/or analyzed during the current study will be available from the corresponding author upon reasonable request.

## Abbreviations

LBW: Low birth weight
*MC4R*: Melanocortin 4 receptor
*MTNR1B*: Melatonin Receptor 1B
*PPAR-γ*: Peroxisome proliferator-activated receptor gamma
*PTEN*: Phosphatase and tensin homolog
*ACACB*: Acetyl-CoA Carboxylase Beta
*PPAR-α*: Peroxisome proliferator-activated receptor-α
EDTA: Ethylenediaminetetraacetic acid
*NRXN3*: Neurexin-3-alpha
*NTRK2*: Neurotrophic Receptor Tyrosine Kinase 2
*PCSK1*: Proprotein convertase 1
*A2BP1*: Ataxin-2 binding protein 1
*TMEM18*: Transmembrane 18 gene
*LXR*: Liver X receptor
*BDNF*: *Brain-derived neurotrophic factor*
*TCF7L2*: Transcription factor 7-like 2
*FTO*: Fat mass and obesity-associated
*CPT1A*: Carnitine palmitoyltransferase IA
PBMC: Peripheral blood mononuclear cells
cDNA: Complementary DNA
